# Target-controlled infusion of remifentanil with or without flurbiprofen axetil in sedation for extracorporeal shock wave lithotripsy of pancreatic stones: a prospective, open-label, randomized controlled trial

**DOI:** 10.1186/s12871-015-0141-6

**Published:** 2015-11-07

**Authors:** Yu-Guang Yang, Liang-Hao Hu, Hui Chen, Bo Li, Xiao-hua Fan, Jin-bao Li, Jia-Feng Wang, Xiao-Ming Deng

**Affiliations:** 1 Department of Anesthesiology and Intensive Care, Changhai Hospital, the Second Military Medical University, 168 Changhai Road, Shanghai, 200433 PR China; 2 Department of Gastroenterology, Changhai Hospital, the Second Military Medical University, Shanghai, 200433 PR China

**Keywords:** Remifentanil, Flurbiprofen axetil, Extracorporeal shock wave lithotripsy, Pancreatic stone, Sedation

## Abstract

**Background:**

Extracorporeal shock wave lithotripsy (ESWL) is an effective therapeutic method used to treat patients with pancreatic stones. However, the anesthesia for this procedure has been underappreciated, with minimal reports of these procedures in certain case series with general or epidural anesthesia.

**Methods:**

A cohort of 60 patients who elected to undergo ESWL in order to treat pancreatic stones for the first time were randomly selected and divided into two groups. One group of patients received target controlled infusion (TCI) of remifentanil, while the other group of patients received TCI of remifentanil plus a bolus of flurbiprofen axetil (a cyclooxygenase inhibitor) (Rem group and Rem + Flu group, *n* = 30 for each group). The Dixon’s up-and-down method was used to calculate the half maximum effective concentration (EC_50_) of remifentanil. Visual analogue scales of pain, Ramsay sedation scale, hemodynamic changes, and adverse events were also recorded.

**Results:**

The EC_50_ of remifentanil was calculated to be 4.0 ng/ml (95 % confidential interval: 3.84 ng/ml, 4.16 ng/ml) and 2.76 ng/ml (95 % confidential interval: 2.63 ng/ml, 2.89 ng/ml) in the Rem group and Rem + Flu group respectively (*p* < 0.001). Pain score was comparable between the two groups, while the Ramsay sedation scale was higher in the Rem group. Hemodynamic data showed that patients in the Rem group experienced higher mean arterial pressures and higher heart rates across the procedures. Patients in Rem group demonstrated a lower respiratory rate (*p* < 0.001) and a lower SpO_2_ (*p* = 0.001). Less adverse events occurred in Rem + Flu group, including a reduced respiratory depression requiring wake-up as well as reduced postoperative nausea and vomiting.

**Conclusion:**

Remifentanil plus flurbiprofen axetil provided satisfactory analgesia and sedation for ESWL of pancreatic stones with less adverse events. (Clinicaltrial.gov: NCT01998217; registered on November 19, 2013)

## Background

Intracanalar or intraductal stones are pathognomonic symptoms of chronic pancreatitis, where these stones tend to cause further obstruction of outflow from the pancreas, ultimately leading to recurrent attacks of pancreatitis and abdominal pain [1, 2]. Extracorporeal shock wave lithotripsy (ESWL) has been used over the past two decades to help treat pancreatic stones, when routine endotherapy cannot be effectively applied [3, 4].

Despite providing pain relief to these patients through ESWL,, the procedure itself is quite painful and requires the addition of anesthesia in a patient’s treatment plan [5]. Moreover, general anesthesia or epidural anesthesia has been utilized for this procedure [5–8], but the elucidation of its effectiveness has infrequently been documented. General anesthesia and epidural anesthesia are known to be invasive procedures due to the involvement of tracheal intubation or epidural puncturing. Both of these processes are time-consuming and require long induction and/or recovery periods.

Monitored anesthesia care with propofol and/or opioids has been clinically applied in the conduction of ESWL of stones in the urinary system, yielding a satisfactory sedation and analgesia results, which is complimented with a shorter recovery time as well [9]. In addition to this, single use of opioids such as remifentanil, fentanyl and sufentanil has also been proposed as an ideal sedation strategy for ESWL of kidney stones [10, 11]. Remifentanil seems to be a more suitable remedy on the grounds that it harbors a lower rate of incidence in events related to respiratory depression and postoperative nausea and vomiting (PONV) [11]. However, there has been a dismal amount of published literature reports describing the quantity at which remifentanil can be considered an effective and safe therapy for ESWL of pancreatic stones. In this study, we sought to report the single use of remifentanil, which was administered through target-controlled infusion (TCI), both in the presence and absence of a cyclooxygenase inhibitor, flurbiprofen axetil, in ESWL of pancreatic stones.

## Methods

### Study design

This was a prospective, open-label, randomized controlled clinical trial. This study was approved by the Committee on Ethics of Biomedicine Research, Second Military Medical University, Shanghai, China. Informed consent was obtained from all patients or a surrogate individual before recruitment into the study.

### Patient inclusion and randomization

The sixty patients enrolled in this study were diagnosed with chronic pancreatitis for the first time, and were treated with ESWL for pain relief in the Center of Gastrointestinal Endoscopy, Changhai Hospital. Patients were enrolled and recruited continuously from September 2012 to December 2013. The patients were randomly allocated into one of two groups, based on an assigned number that was randomly generated on a computer before assignment to the patient. Both groups, Rem group and Rem + Flu group, consisted of thirty individual members (*n* = 30). Randomization was performed by JFW and enrollment of the patients was conducted by YGY and LHH.

The inclusion criteria for ESWL indication were met based on previous reports [12, 13]. Patients undergoing ESWL were excluded from this study, if the following criteria were met: 1) aged ≤18 years or ≥ 65 years; 2) ASA III or higher; 3) patients with hypertension; 4) patients with compromised cardiopulmonary function; 5) patients that have undergone ESWL. Patients would also be excluded in the presence of life-threatening adverse events such as cardiovascular or respiratory failure, as well as mechanical complications induced by the ESWL procedure.

### Trial protocol

After recruitment, patients were instructed to describe their pain based on the use of the visual analogue scale (VAS) (0: no pain; 10: worst possible pain). Electrocardiogram (ECG), heart rate (HR), non-invasive blood pressure (BP), pulse oximetry (SpO_2_) and respiratory rate (RR) were all monitored through the use of the M1106C multi-function monitor (HP, Palo Alto, USA). For sedation induction, 2 L/min of oxygen was inspired and target-controlled infusion of remifentanil (Renfu Pharmaceutical Co., Ltd., Yichang, China) was initiated at the predetermined effect-site by using the Minto model in a Fresenius Vial computer-assisted syringe pump (Fresenius Vial, Grenoble, France). Fluobiprofen axetil (50 mg, Tide Pharmaceutical Co. Ltd., Beijing, China) was administered before the infusion of remifentanil in the Rem + Flur group. ESWL (Compact Delta II, Dornier Med Tech., Wessling, Germany) was initiated 3 min after the effect-site concentration reached a relatively stable level. Shock waves up to a maximum of 5000 shocks were delivered per sitting. An intensity of 6 (16,000 kV) on a scale of 1 to 6 was used with a frequency of 120 shocks/min during the procedure.

The initial concentration of remifentanil was set at 3 ng/ml, and 0.5 ng/ml was chosen as the ideal concentration step for either increment or decrement according to the Dixon’s up-and-down method [14]. A positive response was defined as a VAS score higher than 3 or a complaint of insufferable pain by the patient. In either case, the analgesic effect was considered to be inadequate, and the concentration of remifentanil in the next patient would be increased by 0.5 ng/ml. For the patients that experienced insufferable pain, the concentration of remifentanil was appropriately titrated to a specific level that corresponded to a VAS score lower than 3 to offset insufferable pain. A negative response was defined as one that contained a VAS score that was lower than 3. The concentration of remifentanil in the next set of patients for this case would be decreased by a concentration of 0.5 ng/ml. If the patients were over-sedated, an indication that was detected by a SpO_2_ lower than 91 % or a respiratory rate lower than ten times per minute, then the patients were woken up and were requested to take deep breathes. Ventilation with the facial mask would be initiated, if the patients did not awake or if they could not maintain spontaneous breathing patterns. If an increase in remifentanil was unable to alleviate the unbearable pain associated with the procedure, the patient would be given the opportunity to request for general anesthesia. Remifentanil infusion was terminated after the completion of ESWL, and the patients were transferred to a recovery room and were medically monitored for an additional 30 min.

### Data collection and outcome

Physical signs included: HR, SpO_2_, mean arterial pressure (MAP), and RR, all of which were recorded before induction, after induction, and every 5 min after initiation of ESWL, by HC and JBL, who were blinded to the grouping. VAS score and Ramsay sedation scale (0, does not respond to test stimulus; 1, does not respond mild prodding or shaking; 2, responds only after mild prodding or shaking; 3, responds only after name is called loudly and/or repeatedly; 4, lethargic response to name spoken in normal tone; 5, responds readily to name spoken in a normal tone; 6, agitated) were also assessed every 5 min.

The primary outcome is the half maximal effective concentration (EC_50_) of remifentanil in the two groups. The secondary outcome included VAS score, Ramsay sedation scale, as well as hemodynamic and respiratory parameters. The incidences of adverse effects recorded were: respiratory repression requiring waking up, chest wall rigidity, pruritus, and PONV within the first 24 h after procedure.

### Sample size

The calculation of the sample size for an up-and-down method has not been clearly defined, but a sample size of 20 – 40 for each group is generally considered acceptable [15]. Therefore, a total of 30 patients were included for each group in this study.

### Statistical analysis

All statistical processes were accomplished in SPSS 16.0. The continuous data in normal distribution were expressed as mean ± standard derivation (SD) and those in non-normal distribution were expressed as mean (25 % percentile, 75 % percentile). The EC_50_ of remifentanil was calculated by the isotonic regression estimators, which were proposed in a report by Pace et al. When an order violation was present, the pooled adjacent-violator algorithm was used for adjustment [15–17]. Comparison of the concentrations was accomplished by student’s *t* test following the logarithm transformation. VAS score and Ramsay sedation scale were compared by the Mann–Whitney test. The HR, BP, RR and SpO_2_ were compared in relation to the baseline level by using Student’s *t*-test. Chi-square test was used to compare the incidences of side effects. A *p* < 0.05 was considered to be statistically significant.

## Results

**Fig. 1:**
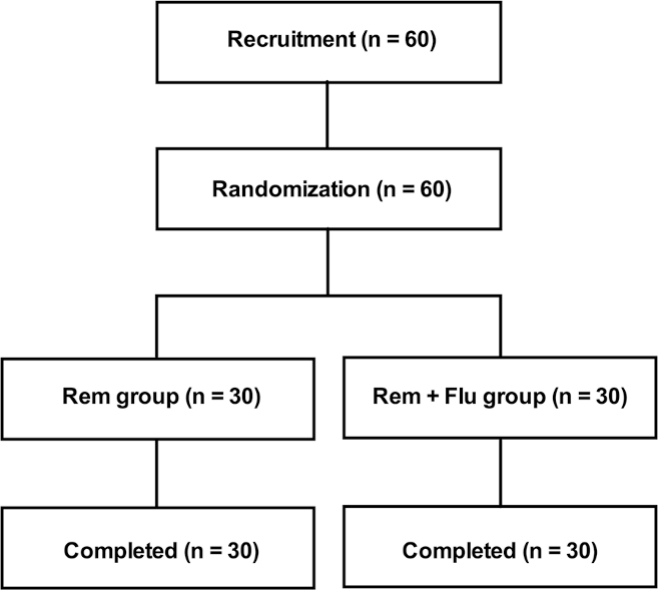
Flow diagram of the recruitment process

All 60 patients completed the study, and no patient ceased participation during the study for safety issues. The patients included in this study were all eligible for data analysis (Fig. 1). The demographic data of the patients are described in Table 1. The age, gender, height, weight, body mass index and procedure duration were similar amongst the two groups.

**Table 1:** Demographic data of the participants

	Rem (*n* = 30)	Rem + Flu (*n* = 30)	*P* value
Age	42.2 ± 11.3	40.7 ± 8.3	0.552
Gender	22/8	19/11	0.405
Height	169.2 ± 7.1	166.3 ± 8.2	0.154
Weight	57.8 ± 8.4	58.3 ± 10.6	0.844
BMI	20.2 ± 2.5	20.9 ± 2.4	0.242
Procedure Duration	55.5 ± 8.1	43.2 ± 6.2	0.217

*BMI* body mass index

**Fig. 2:**
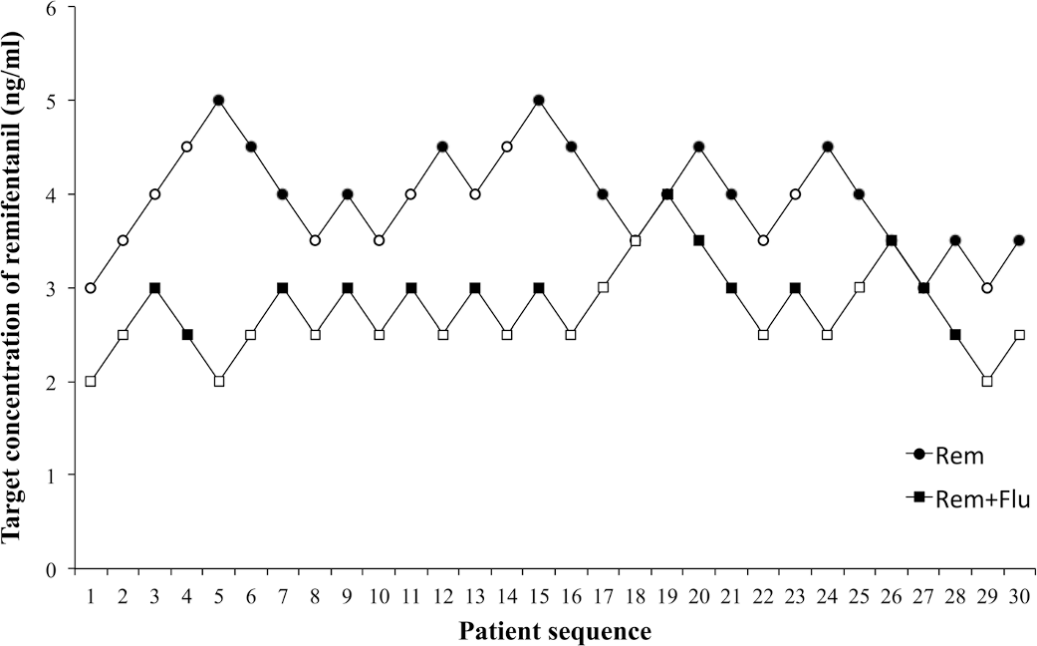
Individual response to ESWL according to the up-and-down sequence by using target-controlled infusion of remifentanil. When a patient showed an increase in either heart rate or mean arterial pressure of 15 % or higher from the pre-ESWL value, the target concentration of remifentanil in the next patient was increased (open symbols). Although neither heart rate nor mean arterial pressure increased by 15 % compared with the pre-ESWL value, the concentration of remifentanil in the next patient was decreased (filled symbols). ESWL, extractoreal shock wave lithotripsy

The hemodynamic changes of the individuals in response to the ESWL procedures are shown in Fig. 2. A negative response led to a 0.5 down-regulation of the remifentanil concentration and vice versa. EC_50_ was calculated by the isotomic regression estimators with adjustment of pooled adjacent-violator algorithm, in both groups. The EC_50_ was 4.0 ng/ml (95 % confidential interval: 3.84 ng/ml, 4.16 ng/ml) and 2.76 ng/ml (95 % confidential interval: 2.63 ng/ml, 2.89 ng/ml) in Rem group and Rem + Flu group respectively. The Student’s *t* test after the logarithm transformation showed that the remifentanil concentration was significantly lower in Rem + Flu group in comparison to the Rem group (*p* < 0.001).

**Fig. 3:**
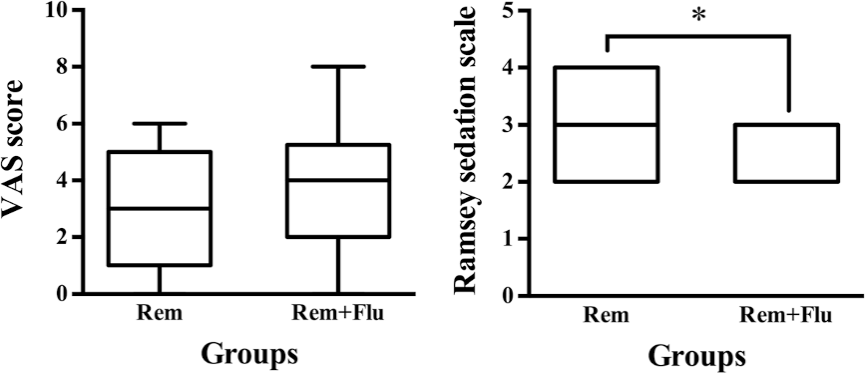
VAS score and Ramsay sedation scale in the two groups (*n* = 30 for both groups). *p = 0.032

**Fig. 4:**
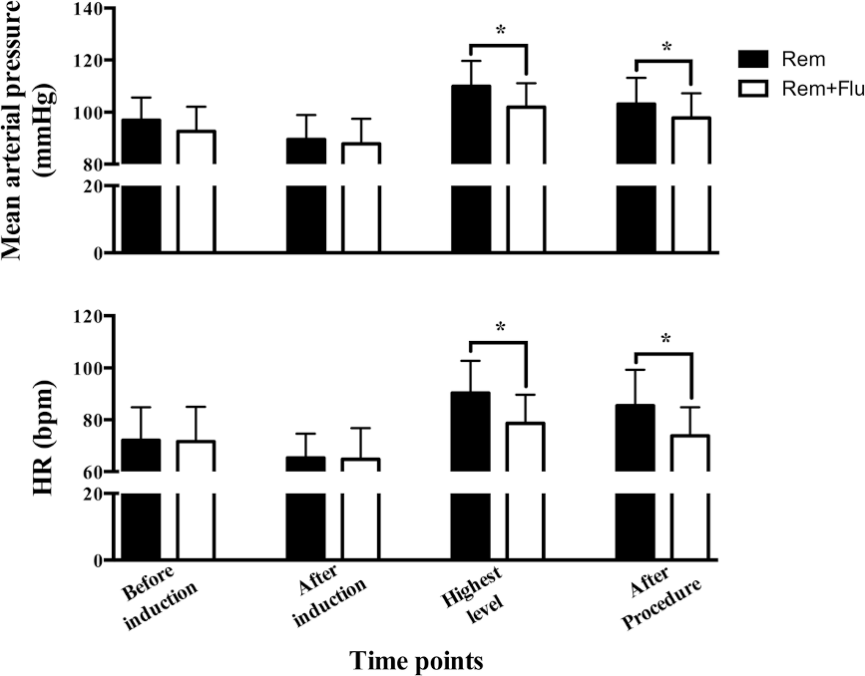
MAP and HR before induction, after induction, at the highest level and after procedure in the two groups (*n* = 30 for both groups). MAP, mean arterial pressure; HR, heart rate. *p < 0.05

**Fig. 5:**
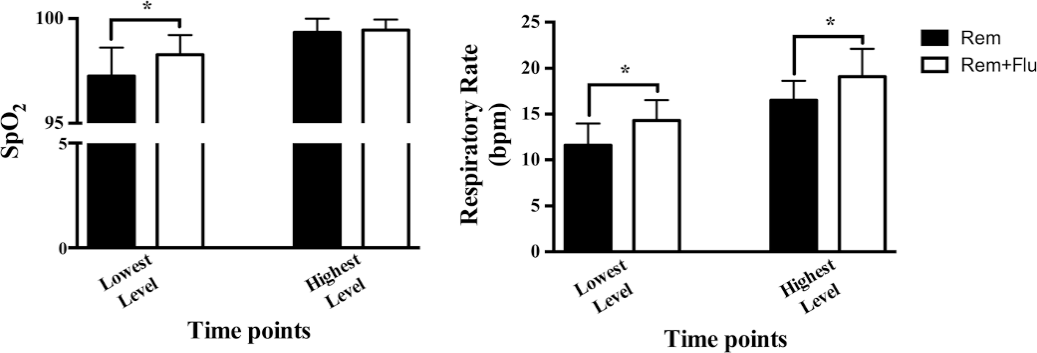
SpO_2_ and RR at the lowest and highest levels in the two groups (*n* = 30 for both groups). RR, respiratory rate. *p < 0.01

The median VAS scores were 3.0 (1.0, 5.0) and 4.0 (2.0, 5.3) in the Rem group and Rem + Flu group (*p* = 0.353). But the Ramsay sedation scale in Rem group was significantly higher [3.0, (2.0, 4.0)] than that in the Rem + Flu group [3.0, (2.0, 3.0)] (*p* = 0.032) (Fig. 3). The hemodynamic data showed that patients in Rem group had higher MAP and HR than the patients in the Rem + Flu group, at both the highest level and after procedure (*p* < 0.05) (Fig. 4). The lowest SpO_2_ in the Rem group is 97.3 ± 0.24 %, slightly lower than the 98.3 ± 0.17 % in Rem + Flu group (*p* = 0.001). The RRs at both the lowest and highest levels were significantly lower in Rem group than in the Rem + Flu group (*p* < 0.001) (Fig. 5).

No severe adverse events occurred during the study. Furthermore, no participants were subjected to assisted ventilation or general anesthesia due to over-sedation and insufficient analgesia. The most common adverse event recorded was PONV, followed by pruritus, and then by respiratory repression requiring waking up. However, incidences of respiratory repression requiring waking up and PONV were significantly lower in the Rem + Flu group than in the Rem group (*p* < 0.05) (Table 2).

**Table 2:** Adverse events associated with the sedation procedure

	Rem (*n* = 30)	Rem + Flu (*n* = 30)	*P* value
Intraoperative wake-up (%)	5 (16.7)	0 (0)	0.020
Chest wall rigidity (%)	0 (0)	0 (0)	1.000
Pruritus (%)	5 (16.7)	2 (6.7)	0.227
PONV (%)	14 (46.7)	8 (26.7)	0.029

*PONV* postoperative nausea and vomiting

## Discussion

This study establishes a novel safe and effective sedation strategy for ESWL of pancreatic stones with remifentanil, in the presence and absence of flurbiprofen axetil. The addition of flurbiprofen axetil was observed to significantly decrease the requirement of remifentanil, while simultaneously promoting a reduction in the side effects of remifentanil, such as respiratory depression and PONV.

ESWL technique has been previously used in treating pancreatic stones over the past three decades, providing satisfactory pain relief for patients and less demand for surgery. Although general anesthesia and epidural anesthesia has been well accepted in certain centers [5–8], few studies have been performed to investigate the suitability of anesthetic technique for pancreatic ESWL. Our present study suggests that a single infusion of remifentanil is an ample amount for analgesia and sedation during ESWL. Furthermore, this single infusion prevented tracheal intubation and epidural puncture. The sedation strategy with remifentanil reduced the induction and recovery time of conventional general or epidural anesthesia, and might be regarded as the standard monitored anesthesia care for ESWL of pancreatic stones.

Sedation and analgesia with remifentanil has been utilized in anesthesia for urinary ESWL [9, 10]. However, the EC_50_ of remifentanil in the Rem group of our study (4.00 ng/ml) was higher than that in anesthesia (2.8 ng/ml) for the ESWL of urinary stones [11]. Thus, the pain induced by ESWL of pancreatic stones might be more severe than that induced by ESWL of urinary stones. The cardiopulmonary inhibition of remifentanil was the primary risk in our study, especially at a high target concentration. Nevertheless, our results did not demonstrate inhibition of circulation by remifentanil during ESWL, even though the target concentration of remifentanil reached 5.0 ng/ml in the two patient populations. Although the respiratory rate was observed to be slower in some patients, the lowest Ramsay sedation scale is 2, and all patients could be roused to avoid respiratory inhibition. Intraoperative body movement was another issue concerned by the gastroenterological physicians.^5^ In this study, we did not observe any body movement during the procedure and no mechanical adverse events were induced while the procedure was conducted.

The addition of flurbiprofen axetil provided several advantages for the remifentanil sedation. Firstly, it reduced the remifentanil amount and prevented over-sedation and respiratory depression, while demonstrating a higher Ramsay sedation scale, higher lowest SpO_2_, as well as less frequency of intraoperative wake-up. Secondly, it improved the inhibition of sympathetic responses that were induced by the ESWL procedures due to a decreased MAP and HR in the Rem + Flu group, at the highest level and after procedure. Less sympathetic responses should be very helpful in patients with high risk of cardiovascular diseases. Thirdly, flurbiprofen axetil also decreased the incidence of PONV, which was induced by remifentanil administration.

PONV is the most common adverse effect of general anesthesia, which is known to possess an incidence rate that is higher than 30 % [18]. In our study, the incidence of PONV was recorded to be 46.7 % in the Rem group and remained as high as 26.7 % in Rem + Flu group, despite a reduction in the concentration of remifentanil. The high incidence of PONV might warrant the use of prophylactic anti-emetic medications, which should be investigated in future trials. Any severe complication induced by postoperative nausea and vomiting, such as: hemorrhage, perforation, or pancreatitis, was not observed.

The main limitation of our study was that it did not compare the sedation strategy with conventional anesthesia, such as general anesthesia or epidural anesthesia. However, we are convinced that the simplified and safe manipulation, as well as the shortened induction and recovery time, would be a more preferable option for patients, anesthesiologist, and gastroenterological physicians.

## Conclusions

In summary, our study demonstrated that monitored anesthesia care with remifentanil provided satisfied sedation and analgesia for ESWL of pancreatic stones. The EC_50_ of remifentanil was 4.0 ng/ml (3.84 ng/ml, 4.16 ng/ml) and 2.76 ng/ml (2.63 ng/ml, 2.89 ng/ml) in the presence or absence of flurbiprofen axetil respectively. The use of flurbiprofen axetil decreased the incidence of respiratory depression and PONV induced by remifentanil, while preventing sympathetic responses that were induced by the procedure, in a more effective fashion. The prophylactic use of anti-emetic medications should be further evaluated in another randomized controlled trial.

## Abbreviations

TCI: Target controlled infusion
ESWL: Extracorporeal shock wave lithotripsy
EC_50_: Half maximum effective concentration
PONV: Postoperative nausea and vomiting
ECG: Electrocardiogram
HR: Heart rate
BP: Blood pressure
SpO_2_: Pulse oximetry
RR: Respiratory rate
VAS: Visual analogue scale
MAP: Mean arterial pressure
SD: Standard derivation
